# New Variable-Weight Optical Orthogonal Codes with Weights 3 to 5

**DOI:** 10.3390/e26110982

**Published:** 2024-11-15

**Authors:** Si-Yeon Pak, Hyo-Won Kim, DaeHan Ahn, Jin-Ho Chung

**Affiliations:** 1 Department of AI Convergence, University of Ulsan, Ulsan 44610, Republic of Korea; tldus86@mail.ulsan.ac.kr (S.-Y.P.); kimhw0914@mail.ulsan.ac.kr (H.-W.K.); 2 Department of Electrical, Electronic, and Computer Engineering, University of Ulsan, Ulsan 44610, Republic of Korea; daehan@ulsan.ac.kr

**Keywords:** multiple access, optical codes, optical networks

## Abstract

In optical networks, designing optical orthogonal codes (OOCs) with appropriate parameters is essential for enhancing the overall system performance. They are divided into two categories, constant-weight OOCs (CW-OOCs) and variable-weight OOCs (VW-OOCs), based on the number of distinct Hamming weights present in their codewords. This paper introduces a method for constructing VW-OOCs of length kp by using the structure of an integer ring and the Chinese Remainder Theorem. In particular, we present some specific VW-OOCs with weights of 3, 4, or 5. The results demonstrate that certain optimal VW-OOCs can be obtained with parameters that are not covered in the existing literature.

## 1. Introduction

Optical networks are advanced communication systems that utilize light to transmit data through optical fiber cables. Known for their high bandwidth and ability to span extensive distances with minimal signal loss, these networks are essential components in the backbone of internet infrastructure, telecommunications, and large-scale enterprise systems. Within this framework, optical orthogonal codes (OOCs) serve a foundational role, enabling code-division multiple access (CDMA) technology. CDMA allows multiple signals to coexist on the same optical channel, reducing interference and enhancing network efficiency by supporting simultaneous transmissions from multiple users without significant cross-talk [1,2,3]. This multiplexing capability not only elevates the data capacity of optical networks but also supports the diverse, high-volume communication demands characteristic of today’s digital landscape. Furthermore, optical networks provide enhanced security and resilience. Their immunity to electromagnetic interference and increased protection against eavesdropping make them particularly valuable for sensitive data transmission in financial, defense, and corporate sectors. OOCs, in this context, function as structured sets of sequences composed of ‘0’ s and ‘1’ s, where ‘1’ signals a light pulse and ‘0’ represents its absence. These codes are meticulously designed to meet specific auto-correlation and cross-correlation criteria, which are critical for minimizing interference between signals. By satisfying these requirements, OOCs enable multiple users to access the network concurrently with reduced signal overlap and interference, thereby ensuring highly efficient and reliable data transmission over optical channels. Research into the development and enhancement of these codes has focused on maximizing clarity and capacity within fiber-optic systems, positioning OOCs as an essential element in advancing the performance and scalability of optical communication networks.

OOCs can be broadly classified into two main categories: constant-weight OOCs (CW-OOCs) and variable-weight OOCs (VW-OOCs), based on the variety of Hamming weights present in their codewords. CW-OOCs are designed to maintain a uniform Hamming weight across all codewords, which typically supports a single quality-of-service (QoS) level. In contrast, VW-OOCs feature codewords with varying Hamming weights, allowing them to accommodate multiple QoS levels within a single framework, making them more versatile for complex multi-user environments. Foundational principles for designing OOCs were established by Chung, Salehi, and Wei [3], who introduced several optimal constructions of CW-OOCs. Their work laid the groundwork for subsequent research, and numerous studies have expanded upon these designs to create CW-OOCs optimized according to the Johnson bound [4], a theoretical limit that defines the maximum achievable codeword family-size under specific correlation constraints [5,6,7,8,9,10]. Building on this foundation, Yang [11] later introduced VW-OOCs, generalizing the Johnson bound to account for codes with variable weights, thus establishing theoretical limits on their sizes. Since Yang’s pioneering work, a range of optimal VW-OOCs with limited weight sets have been developed, meeting the theoretical bounds established in [11] and providing practical solutions for multi-QoS optical communication systems [11,12,13,14,15]. Notably, weights of 1 or 2 are rarely employed in OOC design, as they typically suffer from lower capacity and are more vulnerable to noise in multi-user settings. Consequently, OOCs are generally designed with weights of 3 or higher, balancing robustness and capacity in alignment with the demanding requirements of modern optical networks.

The codes commonly employed in on–off keying (OOK) systems possess several advantageous properties that can be adapted to enhance coherent optical communication systems, even though these systems utilize different modulation techniques. OOCs, for instance, are particularly effective at reducing multi-user interference and mitigating noise—qualities that are crucial in coherent systems, where maintaining signal integrity is paramount. When adapted to function alongside phase and amplitude modulation schemes, such as Quadrature Phase Shift Keying (QPSK) or Quadrature Amplitude Modulation (QAM), OOCs can offer additional robustness against interference, thereby enhancing performance in multi-user coherent environments. Moreover, the inherent time-based structure of OOCs makes them suitable for roles beyond simple data transmission. For instance, they can act as auxiliary signals or error-correction codes, adding layers of signal verification and resilience that are beneficial in the stringent conditions of coherent communications [16]. These versatile applications of OOCs in both traditional and coherent optical systems have driven continued interest and research in OOC designs with both constant and variable weights [17,18,19,20,21], aiming to improve compatibility and performance in increasingly complex optical networks.

Exploring new lengths and weight ratios in VW-OOC design is crucial to address the diverse requirements of modern optical communication systems. Such codes with new parameters enable more flexible designs that can be precisely tailored for specific applications, including long-distance or low-power communication. This adaptability enhances the scalability and efficiency of optical networks, which increasingly demand customized solutions. In this paper, we introduce a construction method for VW-OOCs of length kp, leveraging the structure of an integer ring and the Chinese Remainder Theorem [16]. Specifically, we present VW-OOCs with weights of 3, 4, or 5, which are shown to be optimal with respect to the bound in [13]. This approach not only verifies optimal parameters but also yields new VW-OOCs with configurations not previously covered in the literature. The organization of this paper is as follows: in Section 2, some relevant backgrounds and the foundational framework for the construction of OOCs are presented. In Section 3, a new construction method for VW-OOCs of length kp is provided, and their correlation properties are analyzed. Some fundamental examples to validate the theoretical results and discussion on the parameters are given in Section 4. Finally, some concluding remarks are given in Section 5.

## 2. Backgrounds and Framework

The correlation properties of OOCs play a crucial role in the performance of optical communication systems. Auto-correlation is particularly important for minimizing errors in pattern recognition within noisy environments. Low auto-correlation values at non-zero shifts prevent the significant alignment of a code pattern with itself when displaced, thereby reducing false detection rates and enhancing the robustness of communication. Cross-correlation, on the other hand, measures the level of interference between codes used by different users. In systems where multiple users share the same frequency spectrum, such as in CDMA, low cross-correlation values are essential, as they allow signals from different users to be distinguished clearly, minimizing interference and reducing the bit error rate. Therefore, optimizing both auto-correlation and cross-correlation in OOC design is fundamental to achieving accurate, interference-free data transmission, which significantly enhances the performance and efficiency of optical CDMA systems.

In practice, constructing an OOC requires balancing the length of the codewords, the desired family size, and the permissible maximum correlation value. For CW-OOCs, this balance is relatively straightforward because all codewords share a constant Hamming weight. However, achieving an optimal set for VW-OOCs becomes more complex due to the presence of multiple distinct weights. Our approach leverages the properties of primitive roots in prime fields and the flexibility provided by the Chinese Remainder Theorem, allowing us to construct code sets that meet varying QoS requirements while maintaining robust correlation characteristics. This section provides an overview of the mathematical framework and theoretical principles that underpin our VW-OOC design.

An OOC of length N is a set of codewords in ${\left\{0,1\right\}}^{N}$ for an integer N. Let C denotes an OOC with codewords $C_{i}:{\left\{C_{i}(t)\right\}}_{t=0}^{N-1}$ for 1≤i≤M, where $C_{i}\left(t\right)\in \{0,1\}$. The number M of codewords is called a family size of C. The correlation between two codewords $C_{i}$ and $C_{j}$ is defined as

(1) $$\Lambda_{i,j}\left(\tau \right)=\sum_{t=0}^{N-1}C_{i}\left(t\right)C_{j}\left(t+\tau \right)$$

for 0≤τ≤N−1 and 1≤i,j≤M. When i=j, $\Lambda_{i,j}(\tau )$ is called the auto-correlation of $C_{i}$. Otherwise, it is called the cross-correlation between $C_{i}$ and $C_{j}$. The maximum correlation value λ of C is defined as the maximum among all the auto-correlation values at τ≠0 and all the cross-correlation values between any two distinct codewords.

A CW-OOC C of length N is a set of codewords $C_{i}$ with a constant weight w, that is,

(2) $$\left|\left\{t|C_{i}\left(t\right)=1,\;0\leq t\leq N-1\right\}\right|=w$$

for each 0≤i≤M−1. If the weights of the codewords are not constant, then the OOC is called a variable-weight OOC. In this paper, we deal with construction of a variable-weight OOC. An OOC with length N and a constant weight w is called an (N,w,λ)-OOC if its maximum correlation value is λ. The Johnson bound [4] provides a theoretical bound on the number of codewords in an (N,w,λ)-OOC.

**Theorem** **1**  **(Johnson bound [4]).** *An* (N,w,λ)*-OOC* C *satisfies*

(3) $$\left|C\right|\leq \left\lfloor \frac{1}{w}\left\lfloor \frac{N-1}{w-1}\left\lfloor \cdots \left\lfloor \frac{N-\lambda }{w-\lambda }\right\rfloor \cdots \right\rfloor \right\rfloor \right\rfloor .$$

*If* $\left|C\right|$ *satisfies (1) with equality, then* C *is called an optimal CW-OOC.*

A VW-OOC $V=\left\{V_{1},\ldots ,V_{M}\right\}$ is different from a CW-OOC in the sense that the codewords $V_{i}={\left\{V_{i}(t)\right\}}_{t=0}^{N-1}$ can have variable weights. Assume that $V_{i}$s in V can have a weight in $W=\left\{w_{1},\ldots ,w_{l}\right\}$, and there exists exactly $r_{j}M$ codewords of weight $w_{j}$ for 1≤j≤l. V is called an (N,W,λ,R) VW-OOC if its maximum correlation value is λ, where $W=\left\{r_{1},\ldots ,r_{l}\right\}$. There are some theoretical bounds on the number of codewords in a VW-OOC [11,13]. According to [13], the number of codewords in an (N,W,1,R) VW-OOC is upper-bounded by

(4) $$\left|V\right|\leq \underset{1\leq i\leq l}{\min}\left\lfloor \frac{1}{r_{i}}\left\lfloor r_{i}\left\lfloor \frac{N-1}{\sum_{i=1}^{m}r_{i}w_{i}\left(w_{i}-1\right)}\right\rfloor \right\rfloor \right\rfloor .$$

If a VW-OOC satisfies (4) with equality, it is an optimal VW-OOC.

## 3. Design of Variable-Weight Codes

In an OOC, the maximum correlation value λ=1 indicates that mutual interference in optical network data transmission can be minimized for enhanced efficiency. There are several studies on CW-OOCs and VW-OOCs with λ=1 in the literature [11,12,13,14,15,17,20]. In this section, we present a construction method for VW-OOCs with λ=1 by utilizing the properties of the primitive roots of prime fields.

### 3.1. Construction of VW-OOCs

Let $Z_{p}=\left\{0,1,\ldots ,p-1\right\}$ be the prime field consisting of p elements. If an element α of $Z_{p}$ generates all the elements of $Z_{p}\backslash \{0\}$ by its exponent, it is called a primitive root modulo p. Note that $\alpha^{p-1}=1$ and $\alpha^{u}\neq 1$ for 1≤u≤p−2. By the Chinese Remainder Theorem [22], for any integer k with $\gcd\left(k,p\right)=1$, an integer t with 0≤t≤kp−1 can be uniquely represented as

(5) $$t:=\left(t_{k},t_{p}\right)$$

where $t_{k}=(t\;mod\;k)$ and $t_{p}=(t\;mod\;p)$.

**Construction** **1.**  *Let* p−1=kL *for two positive integers* k *and* L *. Let* $P_{1},P_{2},\ldots ,P_{M}$ *be subsets of* $Z_{k}$ *satisfying* $\left|P_{a}\cap P_{b}\right|$ *for any* 1≤a≠b≤M *, and* $P_{1}\geq P_{2}\geq \ldots \geq P_{M}$ *. For* 1≤a≤M *and* 0≤i≤L−1 *, the support of the binary code* $V_{a,i}={\left\{V_{a,i}(t)\right\}}_{t=0}^{kp-1}$ *of length* kp *can be represented as*

(6) $${supp(V}_{a,i})=\left\{\left(d,\alpha^{dL+i}\right)\in Z_{k}\times Z_{p}:d\in P_{a}\right\}.$$

*Then, the VW-OOC* V *can be constructed as*

(7) $$V=\left\{V_{a,i}\;:\;1\leq a\leq M\;and\;0\leq i\leq L-1\right\}.$$

**Theorem** **2.**  *The set *V* in Construction 1 is a *(kp,W,1, R)*-OOC with set size *ML*, where *R* is the set *$\left\{\left|p_{1}\right|,\left|p_{2}\right|,\ldots ,\left|p_{M}\right|\right\}=\left\{w_{1},\ldots ,w_{m}\right\}$* with *m≤M*, and *$R=\left\{r_{1},\ldots ,r_{m}\right\}$* with *$r_{l}=\left|\left\{a\;:\;\left|p_{a}\right|=w_{l},\;1\leq a\leq M\right\}\right|/M$* for *1≤l≤m.

**Proof.**  The length kp is clear from the definition of $V_{a,i}$. Furthermore, the weight of $V_{a,i}$ is equal to the set size of $P_{a}$ because any element in ${supp(V}_{a,i})$ corresponds to an element in $P_{a}$. In accordance, the sets W and R are determined directly. The auto- and cross-correlation properties are proven in Section 3.2. □

### 3.2. Correlation Properties of VW-OOCs

The correlation $\Lambda_{\left(a,i\right),(b,j)}\left(\tau \right)$ between two codewords $V_{a,i}$ and $V_{b,j}$ can be written as

(8) $$\Lambda_{\left(a,i\right),(b,j)}\left(\tau \right)=\sum_{t=0}^{kp-1}V_{a,i}(t)V_{b,j}(t+\tau \;mod\;kp)$$

where 0≤τ≤kp−1, or equivalently

(9) $$\Lambda_{\left(a,i\right),(b,j)}\left(\tau \right):\Lambda_{\left(a,i\right),(b,j)}\left(\tau_{k},\tau_{p}\right)=\left|\left({supp(V}_{a,i})+\tau \right)\cap {supp(V}_{b,j})\right|$$

with ${supp(V}_{a,i})+\tau :=\left\{x+\tau \;mod\;kp:x\in {supp(V}_{a,i})\right\}$.

#### 3.2.1. Auto-Correlation

Let $\Lambda_{\left(a,i\right),(a,i)}\left(\tau \right)$ be the auto-correlation value of $V_{a,i}$ at τ with 0≤τ≤kp−1. We have


(10) $$\Lambda_{\left(a,i\right),\left(a,i\right)}\left(\tau \right)=\left|\left({supp(V}_{a,i})+\tau \right)\cap {supp(V}_{a,i})\right|.\;$$

Case (i) τ=0. It is clear that


(11) $$\Lambda_{\left(a,i\right),\left(a,i\right)}\left(0\right)=\left|\left\{\left(d,\alpha^{dL+i}\right):d\in P_{a}\right\}\right|=\left|P_{a}\right|.$$

Case (ii) $\tau_{k}\neq 0$ and $\tau_{p}=0$. We also have

$$\Lambda_{\left(a,i\right),\left(a,i\right)}\left(\tau \right)=\Lambda_{\left(a,i\right),\left(a,i\right)}\left(\tau_{k},0\right)$$

(12) $$=\left|\left\{\left(d+\tau_{k},\alpha^{dL+i}\right)\;:d\in P_{a}\right\}\cap \left\{\left(e,\alpha^{eL+i}\right)\;:e\in P_{a}\right\}\right|.\;$$

Note that if $d+\tau_{k}=e$, then $\alpha^{dL+i}\neq \alpha^{eL+i}$ for $\tau_{k}\neq 0$. Thus, $\Lambda_{\left(a,i\right),\left(a,i\right)}\left(\tau_{k},0\right)=0.$ Case (iii) $\tau_{k}=0$ and $\tau_{p}\neq 0$. In this case, there is no case in which both $\left(d,\alpha^{dL+i}\right)$ and $\left(d,\alpha^{dL+i}+\tau_{p}\right)$ are included in ${supp(V}_{a,i})$. Therefore,


(13) $$\Lambda_{\left(a,i\right),\left(a,i\right)}\left(\tau \right)=0.\;$$

Case (iv) $\tau_{k}\neq 0$ and $\tau_{p}\neq 0$.

(14) $$\Lambda_{\left(a,i\right),\left(a,i\right)}\left(\tau \right)=\Lambda_{\left(a,i\right),\left(a,i\right)}\left(\tau_{k},\tau_{p}\right)\;=\left|\left\{\left(d+\tau_{k},\alpha^{dL+i}+\tau_{p}\right)\;:d\in P_{a}\right\}\cap \left\{\left(e,\alpha^{eL+i}\right)\;:e\in P_{a}\right\}\right|\;=\left\{\left(d,e\right)\in P_{a}\times P_{a}:\left(\tau_{k},\tau_{p}\right)=(d-e,\alpha^{eL+i}\left(1-\alpha^{(d-e)L}\right))\right\}\;\leq 1$$

where the last inequality comes from the fact that there exists a unique e satisfying $\alpha^{eL+i}\left(1-\alpha^{(d-e)L}\right)=\tau_{p}$ for a fixed d−e.

By summarizing the results of Cases (i), (ii), (iii), and (iv), we can conclude that the out-of-phase auto-correlation values of $C_{a,i}$ for all 1≤a≤M and 0≤i≤L−1 are upper-bounded by 1.

#### 3.2.2. Cross-Correlation with a=b

Let $\Lambda_{\left(a,i\right),(a,j)}\left(\tau \right)$ be the cross-correlation value between $V_{a,i}$ and $V_{a,j}$ at τ with 0≤τ≤kp−1. We have


(15) $$\Lambda_{\left(a,i\right),\left(a,j\right)}\left(\tau \right)=\left|\left({supp(V}_{a,i})+\tau \right)\cap {supp(V}_{a,j})\right|$$

Case (i) τ=0. We have

(16) $$\Lambda_{\left(a,i\right),\left(a,j\right)}\left(\tau \right)=\left|\left({supp(C}_{a,i})\right)\cap {supp(C}_{a,j})\right|\;=\left|\left\{\left(d,\alpha^{dL+i}\right):d\in P_{a}\right\}\cap \left\{\left(e,\alpha^{eL+j}\right):e\in P_{a}\right\}\right|\;=0$$

because 0≤i≠j≤L−1.

Case (ii) $\tau_{k}\neq 0$ and $\tau_{p}=0$. In a similar way to Case (i),


(17) $$\Lambda_{\left(a,i\right),\left(a,j\right)}\left(\tau \right)=\left|\left({supp(V}_{a,i})+\tau \right)\cap {supp(V}_{a,j})\right|\;=\left|\left\{\left(d+\tau_{k},\alpha^{dL+i}\right):d\in P_{a}\right\}\cap \left\{\left(e,\alpha^{eL+j}\right):e\in P_{a}\right\}\right|\;=0.$$

Case (iii) $\tau_{k}=0$ and $\tau_{p}\neq 0$.

(18) $$\Lambda_{\left(a,i\right),\left(a,j\right)}\left(\tau \right)=\left|\left\{\left(d,\alpha^{dL+i}+\tau_{p}\right)\;:\;d\in P_{a}\right\}\cap \left\{\left(e,\alpha^{eL+j}\right)\;:e\in P_{a}\right\}\right|\;=\left\{d\in P_{a}\;:\tau_{p}=\alpha^{dL+j}\left(1-\alpha^{i-j}\right)\right\}\;\leq 1.$$

because there exists at most one d between 0 and k−1 satisfying $\alpha^{dL+j}=\left(1-\alpha^{i-j}\right)/\tau_{p}$.

Case (iv) $\tau_{p}\neq 0$ and $\tau_{p}\neq 0$. The correlation value can be written as


(19) $$\Lambda_{\left(a,i\right),\left(a,j\right)}\left(\tau \right)=\left|\left\{\left(d,e\right)\in P_{a}\times P_{a}\;:\left(\tau_{k},\tau_{p}\right)=\left(e-d,\alpha^{eL+j}\left(1-\alpha^{\left(e-d\right)L+(j-i)}\right)\right)\right\}\right|.\;$$

If e−d is fixed to $\tau_{k}$, then there is at most one value for e satisfying

(20) $$\alpha^{eL+j}=\frac{\tau_{p}}{\left(1-\alpha^{\tau_{k}L+\left(j-i\right)}\right)},$$

and so $\Lambda_{\left(a,i\right),\left(a,j\right)}\left(\tau \right)\leq 1$.

#### 3.2.3. Cross-Correlation with a≠b

The cross-correlation value $\Lambda_{\left(a,i\right),(b,j)}\left(\tau \right)$ is between $V_{a,i}$ and $V_{b,j}$ at τ and can be expressed as


(21) $$\Lambda_{\left(a,i\right),(b,j)}\left(\tau \right)=\left|\left\{\left(d,e\right)\in P_{a}\times P_{b}\;:\left(\tau_{k},\tau_{p}\right)=\left(e-d,\alpha^{eL+j}\left(1-\alpha^{\left(e-d\right)L+(j-i)}\right)\right)\right\}\right|\;$$

Case (i) $\tau_{k}=0$. We have

(22) $$\Lambda_{\left(a,i\right),\left(a,j\right)}\left(\tau \right)=\left|\left\{\left(d,e\right)\in P_{a}\times P_{b}\;:\left(0,\tau_{p}\right)=\left(e-d,\alpha^{eL+j}\left(1-\alpha^{\left(e-d\right)L+\left(j-i\right)}\right)\right)\right\}\right|\;\leq \left|\left\{\left(d,e\right)\in P_{a}\times P_{b}\;:e=d\right\}\right|\;\leq 1$$

by the fact that $\left|P_{a}\cap P_{b}\right|\leq 1$.

Case (ii) $\tau_{k}\neq 0$. In this case, if e−d is fixed to e−d, then there exists at most one e satisfying


(23) $$\alpha^{eL+j}=\frac{\tau_{p}}{\left(1-\alpha^{\tau_{k}L+\left(j-i\right)}\right)}.$$

From the results of Section 3.2.1, Section 3.2.2 and Section 3.2.3, we can conclude that V is a (kp,W,1, R) VW-OOC consisting of M distinct codewords. The optimality of VW-OOCs with respect to (4) is defined by the number of subsets satisfying the intersection conditions.

## 4. Examples and Optimality

### 4.1. Construction Example for $w\in \left\{3,4,5\right\}$

Let p=11, k=10, and L=1 in Construction 1. We can obtain subsets $P_{1},\ldots ,\;P_{9}$ of $Z_{10}$ as

(24) $$P_{1}=\{1,\;2,\;3,\;4,\;5\},\;P_{2}=\{4,\;7,\;8,\;9\},\;P_{3}=\{1,\;6,\;8\},\;P_{4}=\{2,\;7,\;10\},\;P_{5}=\{3,\;6,\;7\},\;P_{6}=\{0,\;3,\;9\},\;P_{7}=\{0,\;4,\;6\},\;P_{8}=\{0,\;5,\;8\},\;P_{9}=\{5,\;6,\;9\}.$$

It is clear that $\left|P_{a}\cap P_{b}\right|\leq 1$ for all 1≤a≠b≤9. Then, for 1≤a≤9, we can construct OOC $C_{a,0}$ of length 110 with

(25) $${supp(V}_{1,0})=\left\{63,\;65,\;92,\;101,\;104\right\},\;{supp(V}_{2,0})=\left\{7,\;39,\;58,\;104\right\},\;{supp(V}_{3,0})=\left\{58,\;86,\;101\right\},\;{supp(V}_{4,0})=\left\{7,\;92,\;100\right\},\;{supp(V}_{5,0})=\left\{7,\;63,\;86\right\},\;{supp(V}_{6,0})=\left\{39,\;63,\;100\right\},\;{supp(V}_{7,0})=\left\{86,\;100,\;104\right\},\;{supp(V}_{8,0})=\left\{39,\;65,\;86\right\},\;{supp(V}_{9,0})=\left\{58,\;65,\;100\right\},$$

where every support is a subset of $Z_{110}$. The set $V_{1}=\left\{V_{1,0},\ldots ,V_{9,0}\right\}$ becomes a $\left(110,\;\left\{3,4,5\right\},1,\left\{\frac{7}{9},\frac{1}{9},\frac{1}{9}\right\}\right)$ VW-OOC with nine codewords. By (4), we obtain

(26) $$\left|V_{1}\right|\leq \left\lfloor 9\left\lfloor \frac{1}{9}\left\lfloor \frac{109}{\frac{20}{9}+\frac{12}{9}+\frac{42}{9}}\right\rfloor \right\rfloor \right\rfloor =9.$$

Therefore, $V_{1}$ is an optimal VW-OOC with respect to the bound (4).

### 4.2. Construction Example for $w\in \left\{3,5\right\}$

If we change the subsets to

(27) $$P_{1}=\{1,\;2,\;3,\;4,\;5\},\;P_{2}=\{0,\;3,\;6\},\;P_{3}=\{0,\;4,\;7\},\;P_{4}=\{0,\;5,\;9\},\;P_{5}=\{1,\;8,\;9\},\;P_{6}=\{2,\;6,\;9\},\;P_{7}=\{2,\;7,\;8\},\;P_{8}=\{3,\;7,\;9\},\;P_{9}=\{4,\;6,\;8\},\;P_{10}=\{4,\;6,\;8\},$$

then we can obtain another VW-OOC $V_{2}$ with parameter $\left(110,\;\left\{3,5\right\},1,\left\{\frac{9}{10},\frac{1}{10}\right\}\right)$ having 10 codewords. Their support sets are given by

$$\left\{63,\;65,\;92,\;101,\;104\right\},\;\left\{39,\;58,\;101\right\},\;\left\{39,\;86,\;92\right\},\;\left\{7,\;58,\;92\right\},\;\left\{63,\;86,\;100\right\},\;\left\{7,\;39,\;63\right\},\;\left\{58,\;86,\;104\right\},\;\left\{7,\;100,\;104\right\},\;\left\{7,\;65,\;86\right\},\;\left\{39,\;65,\;100\right\}.$$

By (4), we have

(28) $$\left|V_{2}\right|\leq \left\lfloor 10\left\lfloor \frac{1}{10}\left\lfloor \frac{109}{5.4+2}\right\rfloor \right\rfloor \right\rfloor =10.$$

Therefore, $V_{2}$ is an optimal VW-OOC with respect to the bound (4).

### 4.3. Correlation Heatmaps

**Figure 1:**
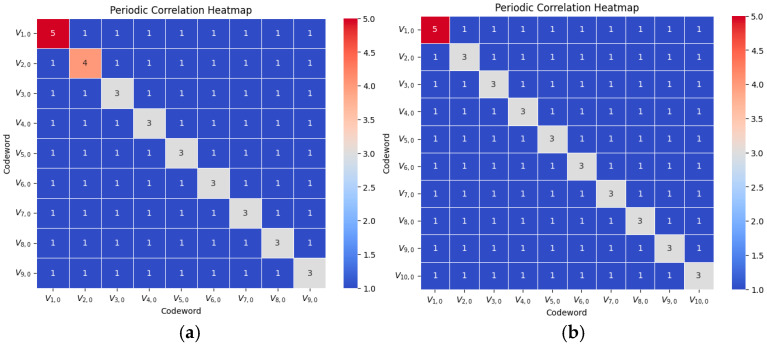
Correlation heatmaps for VW-OOCs (**a**) $V_{1}$ and (**b**) $V_{2}$.

The two heatmaps in Figure 1 represent the maximum correlation values between the two OOCs in $V_{1}$ and $V_{2}$, respectively. In other words, each value is equal to the maximum of $\Lambda_{\left(a,i\right),(b,j)}\left(\tau \right)$ with respect to τ. For example, the correlation between $V_{1,0}$ and $V_{2,0}$ in Section 4.1 is given by

(29) $$\begin{array}{l} {\left\{\Lambda_{\left(1,0\right),\left(2,0\right)}\left(\tau \right)\right\}}_{\tau =0}^{109}\\ =\{1,\;0,\;0,\;0,\;0,\;1,\;0,\;1,\;0,\;0,\;0,\;0,\;0,\;0,\;0,\;0,\;0,\;0,\;0,\;0,\\ 0,\;0,\;0,\;0,\;1,\;0,\;1,\;0,\;0,\;0,\;0,\;0,\;0,\;0,\;1,\;0,\;0,\;0,\;0,\;0,\\ 0,\;0,\;0,\;1,\;0,\;0,\;1,\;0,\;0,\;0,\;0,\;0,\;0,\;1,\;0,\;0,\;1,\;0,\;1,\;0,\\ 0,\;0,\;1,\;0,\;0,\;1,\;0,\;0,\;0,\;1,\;0,\;1,\;0,\;0,\;0,\;0,\;0,\;0,\;0,\;0,\\ 0,\;0,\;0,\;0,\;0,\;1,\;0,\;0,\;0,\;0,\;0,\;0,\;0,\;0,\;1,\;0,\;0,\;1,\;1,\;0,\\ 0,\;0,\;0,\;0,\;0,\;0,\;0,\;1,\;0,\;0\}. \end{array}$$

Thus, the maximum correlation value between $V_{1,0}$ and $V_{2,0}$ is 1.

According to the complete list of correlation values of $V_{1}$ in Appendix A, the maximum correlation between $V_{i,0}$ and $V_{j,0}$ is consistently 1 for every pair, such that 1≤i≠j≤9. This result demonstrates that the codewords exhibit minimal interference with one another, as evidenced by the uniformity of the off-diagonal entries in the heatmap, all of which are equal to 1. This low cross-correlation value is critical for maintaining signal clarity in environments with multiple users, as it ensures that each codeword is distinguishable from the others, thereby reducing interference and enhancing the reliability of multi-user communication. In contrast, the diagonal entries in the heatmap represent the maximum auto-correlation values for each codeword, which vary according to the Hamming weight of the respective codewords. Specifically, these auto-correlation values fall within the range of 3 to 5, depending on the weight, with higher-weighted codewords generally displaying higher auto-correlation peaks. This range in auto-correlation values highlights the distinct design characteristics of each codeword, tailored to achieve optimal performance within the system’s requirements. The variation in these values is integral to the functionality of VW-OOCs, as it enables multiple levels of quality of QoS by assigning different levels of auto-correlation robustness to codewords based on their weights.

Overall, the heatmap effectively visualizes these correlation properties, providing a clear summary of both auto- and cross-correlation properties. The off-diagonal values indicate low cross-interference, while the diagonal entries offer an insight into the internal stability of each codeword. These results support the effectiveness of the proposed VW-OOC design, affirming that the constructed codewords maintain both low auto- and cross-correlation properties (also optimal with respect to (4)), which are essential for efficient and error-resistant data transmission in optical communication systems.

### 4.4. Discussion

The main contributions of previous constructions, along with our new approach for VW-OOCs with a maximum correlation value of 1, are summarized in Table 1. As shown, our construction introduces novel parameters for VW-OOCs with this low correlation threshold, filling gaps that have not been addressed in the existing literature. Unlike previous studies, which primarily focus on specific parameters derived from a fixed formula, our design method for VW-OOCs is notably flexible. Through our approach, optimal parameters can be identified by selecting the appropriate subsets $P_{1},P_{2},\ldots ,P_{M}$ within the framework of Construction 1. This enables a broader parameter search, allowing us to systematically explore configurations that meet the required correlation properties and weight constraints.

Furthermore, by implementing a structured approach to identifying subsets that fulfil these essential correlation and weight conditions, we believe that our methodology could enhance the efficiency and adaptability of VW-OOC construction. The added flexibility in our design process not only enables the generation of VW-OOCs with optimal parameters, but also makes it possible to tailor the codes for diverse applications. This adaptability is particularly valuable for optical communication systems, where varying requirements demand robust, interference-resistant coding solutions.

## 5. Conclusions

A construction method for VW-OOCs of length 𝑘𝑝, utilizing the Chinese Remainder Theorem and the structure of an integer ring, is presented in this paper. In particular, we found some optimal VW-OOCs of length 110, whose weights are between 3 and 5. The results demonstrate the effectiveness of our construction in meeting strict correlation requirements while providing diverse weights suitable for varying QoS levels. In our future works, we plan to conduct further experiments by integrating the designed OOC within practical system environments, such as optical communication setups and multi-user environments, to comprehensively assess its performance in real-world scenarios. Additionally, we aim to broaden the construction framework to derive more flexible parameters from Construction 1, particularly by targeting larger weights and potentially expanding to greater code lengths. By identifying new optimal parameters and extending the construction to accommodate different lengths and weights, we expect to develop an expanded set of VW-OOCs.

## Figures and Tables

**Table 1:** Comparison of parameters of $\left(N,W,1,R\right)$ VW-OOCs.

Reference	Parameters $\left(N,W,1,R\right)$	Conditions
[7]	$\left(p,\left\{w,w+1\right\},1,\left\{\frac{r_{0}}{r_{0}+r_{1}},\frac{r_{1}}{r_{0}+r_{1}}\right\}\right)$	prime $p=w\left(w+1\right)r_{0}+w\left(w-1\right)r_{0}+1$ with odd numbers w
[8]	$\left(lv,\left\{3,4\right\},1,\left\{\frac{1}{2},\frac{1}{2}\right\}\right)$	l=3 or 12, and each prime factor of v is 1 modulo 6
	$\left(6v,\left\{3,4\right\},1,\left\{\frac{1}{2},\frac{1}{2}\right\}\right)$	each prime factor of v is 7 modulo 12
	$\left(6v,\left\{3,6\right\},1,\left\{\frac{1}{2},\frac{1}{2}\right\}\right)$	each prime factor of v is 7 or 31 modulo 36
[9]	$\left(v,\left\{3,4\right\},1,\left\{\frac{1}{2},\frac{1}{2}\right\}\right)$	v≡9 or 45 mod 54
	$\left(v,\left\{4,5\right\},1,\left\{\frac{1}{2},\frac{1}{2}\right\}\right)$	v≡16 or 80 mod 96
[10]	$\left(63v,\left\{3,4,5,6\right\},1,\left\{\frac{1}{19},\frac{6}{19},\frac{6}{19},\frac{6}{19}\right\}\right)$	gcd$\left(v,63\right)=1$, and each prime factor of v is 1 modulo 6
	$\left(105v,\left\{3,4,5,6,7\right\},1,\left\{\frac{1}{25},\frac{6}{25},\frac{6}{25},\frac{6}{25},\frac{6}{25}\right\}\right)$	gcd$\left(v,105\right)=1$, and each prime factor of v is 1 modulo 6
[11]	$\left(19v,\left\{3,4,5\right\},1,\left\{\frac{1}{3},\frac{1}{3},\frac{1}{3}\right\}\right),\;$ $\left(22v,\left\{3,4,5\right\},1,\left\{\frac{1}{2},\frac{1}{4},\frac{1}{4}\right\}\right),\;$ $\left(25v,\left\{3,4,5\right\},1,\left\{\frac{3}{5},\frac{1}{5},\frac{1}{5}\right\}\right),$ $\left(28v,\left\{3,4,5\right\},1,\left\{\frac{2}{5},\frac{2}{5},\frac{1}{5}\right\}\right)$	gcd$\left(v,6\right)=1$
	$\left(24v,\left\{3,4,6\right\},1,\left\{\frac{1}{3},\frac{1}{3},\frac{1}{3}\right\}\right)$, $\left(28v,\left\{3,5,6\right\},1,\left\{\frac{1}{3},\frac{1}{3},\frac{1}{3}\right\}\right)$, $\left(34v,\left\{3,4,5,6\right\},1,\left\{\frac{1}{4},\frac{1}{4},\frac{1}{4},\frac{1}{4}\right\}\right)$	gcd$\left(v,105\right)=1$
This paper	(kp,W,1, R) examples: $\left(110,\;\left\{3,4,5\right\},1,\left\{\frac{7}{9},\frac{1}{9},\frac{1}{9}\right\}\right),$ $\left(110,\;\left\{3,5\right\},1,\left\{\frac{9}{10},\frac{1}{10}\right\}\right)$	k| p−1 W and R depend on the selection of $P_{1},P_{2},\ldots ,P_{M}$.

## Data Availability

No new data were created or analyzed in this study. Data sharing is not applicable (all the numbers in this paper are derived from mathematical structures).

## Appendix A. Correlation Vales of $V_{1}$

**Codeword** **Pair**	Correlation Values (τ= **0~109)**
(1,0), (1,0)	{5,0,1,1,0,0,0,0,0,1,0,0,1,0,0,0,0,0,0,0,0,0,0,0,0,0,0,1,0,1,0,0,0,0,0,0,1,0,1,1,0,1,0,0,0,0,0,0,0,0,0,0,0,0,0,0,0,0,0,0,0,0,0,0,0,0,0,0,0,1,0,1,1,0,1,0,0,0,0,0,0,1,0,1,0,0,0,0,0,0,0,0,0,0,0,0,0,0,1,0,0,1,0,0,0,0,0,1,1,0}
(1,0), (2,0)	{1,0,0,0,0,1,0,1,0,0,0,0,0,0,0,0,0,0,0,0,0,0,0,0,1,0,1,0,0,0,0,0,0,0,1,0,0,0,0,0,0,0,0,1,0,0,1,0,0,0,0,0,0,1,0,0,1,0,1,0,0,0,1,0,0,1,0,0,0,1,0,1,0,0,0,0,0,0,0,0,0,0,0,0,0,1,0,0,0,0,0,0,0,0,1,0,0,1,1,0,0,0,0,0,0,0,0,1,0,0}
(1,0), (3,0)	{1,0,0,1,0,1,1,1,0,0,0,0,0,0,0,1,0,0,1,0,0,0,0,0,0,0,0,0,0,0,0,0,0,0,1,0,0,0,0,0,0,0,0,1,0,0,1,0,0,0,0,0,0,0,0,0,0,0,0,0,0,0,0,0,0,0,0,0,0,0,0,0,1,0,1,0,0,0,0,0,0,0,0,0,0,0,0,1,0,1,0,0,0,0,0,0,0,0,0,0,0,1,0,0,0,0,0,0,0,0}
(1,0), (4,0)	{1,1,0,0,1,0,0,0,0,1,0,0,1,0,0,0,0,0,0,0,0,0,0,0,0,0,0,0,0,0,0,0,0,0,0,0,0,0,0,0,0,0,0,0,0,0,0,0,0,0,0,0,0,0,0,0,1,0,1,0,0,0,0,0,0,0,0,0,0,0,0,0,0,1,0,1,0,0,0,0,0,1,0,1,0,1,0,0,0,0,0,0,0,0,1,0,0,1,0,0,0,0,1,0,0,0,0,0,0,0}
(1,0), (5,0)	{1,0,1,0,0,0,1,0,0,0,0,0,0,0,0,1,0,0,1,0,0,0,0,0,0,0,0,0,0,1,0,0,0,0,0,0,0,0,1,0,0,1,0,0,0,0,0,0,0,0,0,0,0,0,0,0,1,0,1,0,0,0,0,0,0,0,0,0,0,0,0,0,0,0,0,0,0,0,0,0,0,0,0,0,0,1,0,1,0,1,0,0,0,0,1,0,0,1,0,0,0,0,0,0,0,0,0,0,0,0}
(1,0), (6,0)	{1,1,1,0,1,0,0,0,0,0,0,0,0,0,0,0,0,0,0,0,0,0,0,0,1,0,1,0,0,1,0,0,0,0,0,0,0,0,1,0,0,1,0,0,0,0,0,0,0,0,0,0,0,1,0,0,0,0,0,0,0,0,1,0,0,1,0,0,0,0,0,0,0,1,0,1,0,0,0,0,0,0,0,0,0,0,0,0,0,0,0,0,0,0,0,0,0,0,0,0,0,0,1,0,0,0,0,0,0,0}
(1,0), (7,0)	{1,1,0,0,1,0,1,0,0,0,0,0,0,0,0,1,0,0,1,0,0,0,0,0,0,0,0,0,0,0,0,0,0,0,0,0,0,0,0,0,0,0,0,0,0,0,0,0,0,0,0,0,0,0,0,0,0,0,0,0,0,0,0,0,0,0,0,0,0,1,0,1,0,1,0,1,0,0,0,0,0,0,0,0,0,0,0,1,0,1,0,0,0,0,0,0,0,0,1,0,0,0,1,0,0,0,0,1,0,0}
(1,0), (8,0)	{1,0,0,0,0,0,1,0,0,0,0,0,0,0,0,1,0,0,1,0,0,0,0,0,1,0,1,1,0,0,0,0,0,0,0,0,1,0,0,1,0,0,0,0,0,0,0,0,0,0,0,0,0,1,0,0,0,0,0,0,0,0,1,0,0,1,0,0,0,0,0,0,0,0,0,0,0,0,0,0,0,0,0,0,0,0,0,1,0,1,0,0,0,0,0,0,0,0,0,0,0,0,0,0,0,0,0,0,1,0}
(1,0), (9,0)	{1,1,0,0,1,1,0,1,0,0,0,0,0,0,0,0,0,0,0,0,0,0,0,0,0,0,0,1,0,0,0,0,0,0,1,0,1,0,0,1,0,0,0,1,0,0,1,0,0,0,0,0,0,0,0,0,0,0,0,0,0,0,0,0,0,0,0,0,0,0,0,0,0,1,0,1,0,0,0,0,0,0,0,0,0,0,0,0,0,0,0,0,0,0,0,0,0,0,0,0,0,0,1,0,0,0,0,0,1,0}
(2,0), (2,0)	{4,0,0,0,0,0,0,0,0,0,0,0,0,1,0,0,0,0,0,1,0,0,0,0,0,0,0,0,0,0,0,0,1,0,0,0,0,0,0,0,0,0,0,0,0,1,1,0,0,0,0,1,0,0,0,0,0,0,0,1,0,0,0,0,1,1,0,0,0,0,0,0,0,0,0,0,0,0,1,0,0,0,0,0,0,0,0,0,0,0,0,1,0,0,0,0,0,1,0,0,0,0,0,0,0,0,0,0,0,0}
(2,0), (3,0)	{1,0,0,1,0,0,0,0,0,0,0,0,0,0,0,0,1,0,1,0,0,0,0,0,0,0,0,0,0,0,0,1,0,0,0,0,0,0,0,0,0,0,0,0,0,0,1,0,1,0,0,0,0,0,0,0,0,0,0,1,0,0,0,1,0,0,0,1,0,0,0,0,0,0,0,0,0,0,0,0,0,0,1,0,0,0,0,0,0,0,0,1,0,0,0,0,0,0,0,0,0,0,0,0,0,0,0,0,0,0}
(2,0), (4,0)	{1,0,0,0,1,0,0,0,0,0,0,0,1,0,0,0,0,1,0,0,0,0,0,0,0,1,0,0,0,0,0,0,1,0,0,0,0,0,0,0,0,0,0,0,0,0,0,0,0,1,0,1,0,0,0,0,0,1,0,0,0,0,0,0,0,0,0,0,1,0,0,0,0,0,0,0,1,0,0,0,0,0,0,0,0,0,0,0,0,0,0,0,0,0,0,0,0,1,0,0,0,0,0,0,0,0,0,0,0,0}
(2,0), (5,0)	{1,0,0,0,0,0,0,0,0,0,0,0,0,0,0,0,0,0,1,0,0,0,0,0,0,0,0,0,0,0,0,1,1,0,0,0,0,0,0,0,0,1,0,0,0,0,0,0,0,0,0,1,0,0,1,0,0,0,0,0,0,0,0,1,0,0,0,0,0,0,0,0,0,0,0,0,0,0,0,0,0,0,1,0,0,0,1,0,0,0,0,0,0,0,0,0,0,1,0,0,0,0,0,0,0,1,0,0,0,0}
(2,0), (6,0)	{1,0,0,0,1,0,0,0,0,0,0,0,0,0,0,0,0,1,0,1,0,0,0,0,0,0,0,0,0,0,0,0,0,0,0,0,0,0,0,0,0,1,0,0,0,0,0,0,0,1,0,0,0,0,1,0,0,0,0,0,0,0,0,0,0,1,0,0,1,0,0,0,0,0,0,0,0,0,1,0,0,0,0,0,0,0,1,0,0,0,0,0,0,0,0,0,0,0,0,0,0,0,0,0,0,1,0,0,0,0}
(2,0), (7,0)	{1,0,0,0,1,0,0,0,0,0,0,0,0,1,0,0,0,1,1,0,0,0,0,0,0,0,0,0,0,0,0,1,0,0,0,0,0,0,0,0,0,0,0,0,0,1,0,0,0,1,0,0,0,0,0,0,0,0,0,0,0,0,0,1,1,0,0,0,1,0,0,0,0,0,0,0,0,0,0,0,0,0,1,0,0,0,0,0,0,0,0,0,0,0,0,0,0,0,0,0,0,0,0,0,0,0,0,0,0,0}
(2,0), (8,0)	{1,0,0,0,0,0,0,0,0,0,0,0,0,0,0,0,0,0,1,1,0,0,0,0,0,0,0,0,0,0,0,1,0,0,0,0,0,0,0,1,0,0,0,0,0,0,0,0,0,0,0,0,1,0,0,0,0,0,0,0,0,0,0,1,0,1,0,0,0,0,0,0,0,0,0,0,0,0,1,0,0,0,1,0,1,0,0,0,0,0,0,0,0,0,0,0,0,0,0,0,0,0,0,1,0,0,0,0,0,0}
(2,0), (9,0)	{1,0,0,0,1,0,0,0,0,0,0,0,0,0,0,0,0,1,0,0,0,0,0,0,0,0,0,0,0,0,0,0,0,0,0,0,0,0,0,1,0,0,0,0,0,0,1,0,0,1,0,0,1,0,0,0,0,0,0,1,0,0,0,0,0,0,0,0,1,0,0,0,0,0,0,0,0,0,0,0,0,0,0,0,1,0,0,0,0,0,0,1,0,0,0,0,0,0,0,0,0,0,0,1,0,0,0,0,0,0}
(3,0), (3,0)	{3,0,0,0,0,0,0,0,0,0,0,0,0,0,0,1,0,0,0,0,0,0,0,0,0,0,0,0,1,0,0,0,0,0,0,0,0,0,0,0,0,0,0,1,0,0,0,0,0,0,0,0,0,0,0,0,0,0,0,0,0,0,0,0,0,0,0,1,0,0,0,0,0,0,0,0,0,0,0,0,0,0,1,0,0,0,0,0,0,0,0,0,0,0,0,1,0,0,0,0,0,0,0,0,0,0,0,0,0,0}
(3,0), (4,0)	{0,1,0,0,0,0,0,0,0,1,0,0,0,0,0,0,0,0,0,0,0,0,0,0,0,0,0,0,0,0,0,0,0,0,0,0,0,0,0,0,0,0,0,0,0,0,0,0,0,0,0,1,0,0,0,0,0,0,0,0,0,0,0,0,0,0,0,0,1,0,0,0,0,0,0,0,1,0,0,1,0,0,0,0,0,0,0,0,0,0,0,0,0,0,1,0,1,0,0,0,0,0,0,0,1,0,0,0,0,0}
(3,0), (5,0)	{1,0,0,0,0,0,0,0,0,0,0,0,0,0,0,1,0,0,0,0,0,0,0,1,0,0,0,0,0,0,0,0,0,0,0,0,0,0,1,0,0,0,0,0,0,0,0,0,0,0,0,1,0,0,0,0,0,0,0,0,0,0,0,0,0,0,0,0,0,0,0,0,0,0,0,0,0,0,0,1,0,0,1,0,0,0,0,0,0,0,0,0,0,0,1,0,0,0,0,0,0,0,0,0,0,1,0,0,0,0}
(3,0), (6,0)	{0,1,0,0,0,0,0,0,0,0,0,0,0,0,0,0,0,0,0,1,0,0,0,1,0,0,0,0,0,0,0,0,0,0,0,0,0,0,1,0,0,0,0,0,0,0,0,1,0,0,0,0,0,0,0,0,0,0,0,0,0,0,1,0,0,0,0,0,1,0,0,0,0,0,0,0,0,0,0,0,0,0,0,0,0,0,0,0,0,0,0,0,0,0,0,0,1,0,0,0,0,0,0,0,0,1,0,0,0,0}
(3,0), (7,0)	{1,1,0,0,0,0,0,0,0,0,0,0,0,0,0,1,0,0,0,0,0,0,0,0,0,0,0,0,0,0,0,0,0,0,0,0,0,0,0,0,0,0,0,0,0,0,0,0,0,0,0,0,0,0,0,0,0,0,0,0,0,0,0,0,1,0,0,0,1,0,0,0,0,0,0,0,0,0,0,0,0,0,1,0,0,0,0,0,0,0,0,0,1,0,0,0,1,0,0,0,0,0,0,0,0,0,0,1,0,0}
(3,0), (8,0)	{1,0,0,0,0,0,0,0,0,0,0,0,0,0,0,1,0,0,0,1,0,1,0,0,0,0,0,0,0,0,0,0,0,0,0,0,1,0,0,0,0,0,0,0,0,0,0,1,0,0,0,0,0,0,0,0,0,0,0,0,0,0,1,0,0,0,0,0,0,0,0,0,0,0,0,0,0,0,0,0,0,0,1,0,0,0,0,0,0,0,0,0,0,0,0,0,0,0,0,0,0,0,0,1,0,0,0,0,0,0}
(3,0), (9,0)	{1,1,0,0,0,0,0,0,0,0,0,0,0,0,0,0,0,0,0,0,0,1,0,0,0,0,0,0,1,0,0,0,0,0,0,0,1,0,0,0,0,0,0,1,0,0,0,0,0,0,0,0,0,0,0,0,0,0,0,0,0,0,0,0,0,0,0,0,1,0,0,0,0,0,0,0,0,0,0,0,0,0,0,0,0,0,0,0,0,0,0,0,0,0,0,0,1,0,0,0,0,0,0,1,0,0,0,0,0,0}
(4,0), (4,0)	{3,0,0,0,0,0,0,0,1,0,0,0,0,0,0,0,0,1,0,0,0,0,0,0,0,1,0,0,0,0,0,0,0,0,0,0,0,0,0,0,0,0,0,0,0,0,0,0,0,0,0,0,0,0,0,0,0,0,0,0,0,0,0,0,0,0,0,0,0,0,0,0,0,0,0,0,0,0,0,0,0,0,0,0,0,1,0,0,0,0,0,0,0,1,0,0,0,0,0,0,0,0,1,0,0,0,0,0,0,0}
(4,0), (5,0)	{1,0,0,0,0,0,1,0,0,0,0,0,0,0,1,0,0,0,0,0,0,0,0,0,0,0,0,0,0,1,0,1,0,0,0,0,0,1,0,0,0,0,0,0,0,0,0,0,0,0,0,0,0,0,1,0,0,0,0,0,0,0,0,0,0,0,0,0,0,0,0,0,0,0,0,0,0,0,0,0,0,0,0,0,0,1,0,0,0,0,0,0,0,1,0,0,0,0,0,0,0,0,0,0,0,0,0,0,0,0}
(4,0), (6,0)	{1,0,0,0,0,0,0,0,0,0,0,0,0,0,0,0,0,1,0,0,0,0,0,0,0,0,0,0,0,1,0,0,0,0,0,0,0,1,0,0,0,0,0,0,0,0,0,0,0,0,0,0,0,1,1,0,0,0,0,0,0,1,0,0,0,0,0,0,0,0,0,0,0,0,0,0,0,0,1,0,0,0,0,0,0,0,0,0,0,0,0,0,0,0,0,0,0,0,0,0,0,0,1,0,0,0,0,0,0,0}
(4,0), (7,0)	{1,0,0,0,0,0,1,0,0,0,0,0,0,1,1,0,0,1,0,0,0,0,0,0,0,0,0,0,0,0,0,1,0,0,0,0,0,0,0,0,0,0,0,0,0,0,0,0,0,0,0,0,0,0,0,0,0,0,0,0,0,0,0,0,0,0,0,0,0,0,0,0,0,0,0,0,0,0,0,0,0,0,0,0,0,0,0,0,0,0,0,0,0,0,0,0,0,0,1,0,0,0,1,0,0,0,1,0,0,0}
(4,0), (8,0)	{0,0,0,0,0,0,1,0,0,0,0,0,0,0,1,0,0,0,0,0,0,0,0,0,0,0,0,1,0,0,0,1,0,0,0,1,0,0,0,0,0,0,0,0,0,0,0,0,0,0,0,0,1,1,0,0,0,0,0,0,0,1,0,0,0,0,0,0,0,0,0,0,0,0,0,0,0,0,1,0,0,0,0,0,0,0,0,0,0,0,0,0,0,0,0,0,0,0,0,0,0,0,0,0,0,0,0,0,0,0}
(4,0), (9,0)	{1,0,0,0,0,0,0,0,0,0,0,0,0,0,0,0,0,1,0,0,0,0,0,0,0,0,0,1,0,0,0,0,0,0,1,1,0,0,0,0,0,0,1,0,0,0,0,0,0,0,0,0,1,0,0,0,0,0,0,1,0,0,0,0,0,0,0,0,0,0,0,0,0,0,0,0,0,0,0,0,0,0,0,0,0,0,0,0,0,0,0,0,0,0,0,0,0,0,0,0,0,0,1,0,0,0,0,0,0,0}
(5,0), (5,0)	{3,0,0,0,0,0,0,0,0,0,0,0,0,0,0,0,0,0,0,0,0,0,0,1,0,0,0,0,0,0,0,1,0,0,0,0,0,0,0,0,0,0,0,0,0,0,0,0,0,0,0,0,0,0,1,0,1,0,0,0,0,0,0,0,0,0,0,0,0,0,0,0,0,0,0,0,0,0,0,1,0,0,0,0,0,0,0,1,0,0,0,0,0,0,0,0,0,0,0,0,0,0,0,0,0,0,0,0,0,0}
(5,0), (6,0)	{1,0,0,0,0,0,0,0,0,0,0,0,0,0,0,0,0,1,0,0,0,0,0,1,1,0,0,0,0,0,0,0,0,0,0,0,0,0,0,0,0,0,0,0,0,0,0,1,0,0,0,0,0,0,1,0,0,0,0,0,0,0,0,0,0,0,0,0,0,0,0,0,0,1,0,0,0,0,1,0,0,0,0,0,0,0,0,0,0,0,0,0,0,0,0,0,1,0,0,0,0,0,0,0,0,0,0,0,0,0}
(5,0), (7,0)	{1,0,0,0,0,0,0,0,0,0,0,0,0,1,0,0,0,1,0,0,0,0,0,0,0,0,0,0,0,0,0,1,0,0,0,0,0,0,0,0,0,0,0,0,0,0,0,0,0,0,0,0,0,0,0,0,0,0,0,0,0,0,0,0,0,0,0,0,0,1,0,0,0,1,0,0,0,0,0,0,0,0,0,0,0,0,0,1,0,0,0,0,1,0,0,0,1,0,0,0,0,0,0,0,0,0,0,0,0,0}
(5,0), (8,0)	{1,0,0,0,0,0,0,0,0,0,0,0,0,0,0,0,0,0,0,0,0,1,0,0,1,0,0,0,0,0,0,1,0,0,0,0,0,0,0,0,0,0,0,0,0,0,0,1,0,0,0,0,1,0,0,0,0,0,0,0,0,0,0,0,0,0,0,0,0,0,0,0,0,0,0,0,0,0,1,0,0,0,0,0,0,0,0,1,0,0,0,0,0,0,0,0,0,0,0,0,0,0,0,0,0,0,0,0,1,0}
(5,0), (9,0)	{0,0,0,0,0,1,0,0,0,0,0,0,0,0,0,0,0,1,0,0,0,1,0,0,0,0,0,0,1,0,0,0,0,0,0,0,0,0,0,0,0,0,0,0,0,0,0,0,0,0,0,0,1,0,0,0,0,0,0,1,0,0,0,0,0,0,0,0,0,0,0,0,0,1,0,0,0,0,0,0,0,0,0,0,0,0,0,0,0,0,0,0,0,0,0,0,1,0,0,0,0,0,0,0,0,0,0,0,1,0}
(6,0), (6,0)	{3,0,0,0,0,0,0,0,0,0,0,0,0,0,0,0,0,0,0,0,0,0,0,0,1,0,0,0,0,0,0,0,0,0,0,0,0,1,0,0,0,0,0,0,0,0,0,0,0,1,0,0,0,0,0,0,0,0,0,0,0,1,0,0,0,0,0,0,0,0,0,0,0,1,0,0,0,0,0,0,0,0,0,0,0,0,1,0,0,0,0,0,0,0,0,0,0,0,0,0,0,0,0,0,0,0,0,0,0,0}
(6,0), (7,0)	{1,0,0,0,0,0,0,0,0,0,0,0,0,0,1,0,0,0,0,0,0,0,0,0,0,0,0,0,0,0,0,0,0,0,0,0,0,0,0,0,0,0,0,0,0,1,0,0,0,1,0,0,0,0,0,0,0,0,0,0,0,0,0,1,0,0,0,0,0,1,0,0,0,1,0,0,0,0,0,0,0,0,0,0,0,0,0,1,0,0,0,0,0,0,0,0,0,0,0,0,0,0,0,0,0,0,1,0,0,0}
(6,0), (8,0)	{1,0,0,0,0,0,0,0,0,0,0,0,0,0,1,0,0,0,0,0,0,0,0,0,1,0,0,0,0,0,0,0,0,0,0,1,0,0,0,0,0,0,0,0,0,0,0,0,0,0,0,0,0,0,0,0,0,0,0,0,0,1,0,1,0,0,0,0,0,0,0,0,0,0,0,0,0,0,0,0,0,0,0,0,1,0,0,1,0,0,0,0,0,0,0,0,0,0,0,0,0,0,0,0,0,0,0,0,1,0}
(6,0), (9,0)	{1,0,0,0,0,1,0,0,0,0,0,0,0,0,0,0,0,0,0,0,0,0,0,0,0,0,0,0,0,0,0,0,0,0,0,1,0,0,0,0,0,0,1,0,0,0,0,0,0,1,0,0,0,0,0,0,0,0,0,0,0,0,0,0,0,0,0,0,0,0,0,0,0,1,0,0,0,0,0,0,0,0,0,0,1,0,0,0,0,0,0,1,0,0,0,0,0,0,0,0,0,0,0,0,0,0,0,0,1,0}
(7,0), (7,0)	{3,0,0,0,1,0,0,0,0,0,0,0,0,0,1,0,0,0,1,0,0,0,0,0,0,0,0,0,0,0,0,0,0,0,0,0,0,0,0,0,0,0,0,0,0,0,0,0,0,0,0,0,0,0,0,0,0,0,0,0,0,0,0,0,0,0,0,0,0,0,0,0,0,0,0,0,0,0,0,0,0,0,0,0,0,0,0,0,0,0,0,0,1,0,0,0,1,0,0,0,0,0,0,0,0,0,1,0,0,0}
(7,0), (8,0)	{1,0,0,0,0,0,0,0,0,0,0,0,0,0,1,0,0,0,1,0,0,1,0,0,0,0,0,0,0,0,0,0,0,0,0,1,0,0,0,1,0,0,0,0,0,0,0,1,0,0,0,0,0,0,0,0,0,0,0,0,0,1,0,0,0,1,0,0,0,0,0,0,0,0,0,0,0,0,0,0,0,0,0,0,0,0,0,0,0,0,0,0,0,0,0,0,0,0,0,0,0,0,0,0,0,0,0,0,0,0}
(7,0), (9,0)	{1,0,0,0,1,0,0,0,0,0,0,0,0,0,0,0,0,0,0,0,0,1,0,0,0,0,0,0,1,0,0,0,0,0,0,1,0,0,0,1,0,0,1,0,0,0,1,0,0,0,0,0,0,0,0,0,0,0,0,0,0,0,0,0,0,0,0,0,0,0,0,0,0,0,0,0,0,0,0,0,0,0,0,0,0,0,0,0,0,0,0,0,0,0,0,0,1,0,0,0,0,0,0,0,0,0,0,0,0,0}
(8,0), (8,0)	{3,0,0,0,0,0,0,0,0,0,0,0,0,0,0,0,0,0,0,0,0,1,0,0,0,0,1,0,0,0,0,0,0,0,0,0,0,0,0,0,0,0,0,0,0,0,0,1,0,0,0,0,0,0,0,0,0,0,0,0,0,0,0,1,0,0,0,0,0,0,0,0,0,0,0,0,0,0,0,0,0,0,0,0,1,0,0,0,0,1,0,0,0,0,0,0,0,0,0,0,0,0,0,0,0,0,0,0,0,0}
(8,0), (9,0)	{1,0,0,0,0,0,0,1,0,0,0,0,0,0,0,0,0,0,0,0,0,1,0,0,0,0,0,0,1,0,0,0,0,0,0,0,0,0,0,0,0,0,0,0,0,0,0,0,0,1,0,0,0,0,0,0,0,0,0,0,0,0,0,0,0,0,0,0,0,0,0,0,0,0,0,1,0,0,0,0,0,0,0,0,1,0,0,0,0,0,0,1,0,0,0,0,1,0,0,0,0,0,0,0,0,0,0,0,0,0}
(9,0), (9,0)	{3,0,0,0,0,0,0,1,0,0,0,0,0,0,0,0,0,0,0,0,0,0,0,0,0,0,0,0,0,0,0,0,0,0,0,1,0,0,0,0,0,0,1,0,0,0,0,0,0,0,0,0,0,0,0,0,0,0,0,0,0,0,0,0,0,0,0,0,1,0,0,0,0,0,0,1,0,0,0,0,0,0,0,0,0,0,0,0,0,0,0,0,0,0,0,0,0,0,0,0,0,0,0,1,0,0,0,0,0,0}
